# Diagnostic Performance of Dengue NS1 and Antibodies by Serum Concentration Technique

**DOI:** 10.3390/tropicalmed8020117

**Published:** 2023-02-14

**Authors:** Viravarn Luvira, Charin Thawornkuno, Saranath Lawpoolsri, Narin Thippornchai, Chatnapa Duangdee, Thundon Ngamprasertchai, Pornsawan Leaungwutiwong

**Affiliations:** 1 Department of Clinical Tropical Medicine, Faculty of Tropical Medicine, Mahidol University, Bangkok 10400, Thailand; 2 Department of Molecular Tropical Medicine and Genetics, Faculty of Tropical Medicine, Mahidol University, Bangkok 10400, Thailand; 3 Department of Tropical Hygiene, Faculty of Tropical Medicine, Mahidol University, Bangkok 10400, Thailand; 4 Department of Microbiology and Immunology, Faculty of Tropical Medicine, Mahidol University, Bangkok 10400, Thailand; 5 Hospital for Tropical Diseases, Faculty of Tropical Medicine, Mahidol University, Bangkok 10400, Thailand

**Keywords:** dengue, NS1, ultrafiltration, diagnostics

## Abstract

Dengue infection has been a public health problem worldwide, especially in tropical areas. A lack of sensitive diagnostic methods in the early phase of the illness is one of the challenging problems in clinical practices. We, herein, analyzed 86 sera of acute febrile patients, from both dengue and non-dengue febrile illness, to study the diagnostic performance of dengue diagnostics. When compared with detection by Polymerase Chain Reaction (PCR), dengue NS1 detection by enzyme-linked immunosorbent assay (ELISA) had the highest sensitivity of 82.4% (with 94.3% specificity), while NS1 by rapid diagnostic test (RDT) had 76.5% sensitivity. IgM detection by ELISA and RDT showed only 27.5% and 17.9% sensitivity, respectively. The combination of NS1 and IgM in RDT yielded a sensitivity of 78.4%, with 97.1% specificity. One of the essential steps in making a diagnosis from patient samples is the preparation process. At present, a variety of techniques have been used to increase the number of analytes in clinical samples. In this study, we focused on the sample concentration method. The sera were concentrated three times with the ultrafiltration method using a 10 kDa molecular weight cut-off membrane. The results showed an increase in the sensitivity of RDT-NS1 detection at 80.4%, with 100% specificity. When combining NS1 and IgM detection, the concentration method granted RDT an 82.4% sensitivity, with 100% specificity. In conclusion, serum concentration by the ultrafiltration method is a simple and applicable technique. It could increase the diagnostic performance of point-of-care dengue diagnostics.

## 1. Introduction

Dengue is a mosquito-borne disease caused by infection with dengue virus (DENV) serotypes 1–4, which belong to the family *Flaviviridae*, genus *Flavivirus*. Dengue has been a major global public health problem for decades. The disease trend indicates a global rise in both incidence and mortality rate [1]. Dengue has a wide range of clinical presentations and complications as well as an unpredictable clinical course. The clinical spectrum markedly varies and includes asymptomatic, undifferentiated febrile illness, dengue fever (DF), dengue hemorrhagic fever (DHF), which is characterized by plasma leakage and hemorrhagic manifestation, and dengue shock syndrome (DSS). The recent classification includes dengue without and with warning signs and severe dengue. The classical clinical course starts with high-grade fever and non-specific symptoms lasting for 3–7 days during the “febrile phase”, followed by a “defervescence” or “critical phase”, which usually lasts 24–48 h, and a “recovery phase” [2]. To date, there is no specific antiviral treatment for dengue; thus, early recognition and prompt supportive treatment are important for preventing complications, such as bleeding and shock. Early recognition is based on early diagnosis; however, there are still challenges in the diagnostics of dengue. The practical point-of-care tests still pose a limitation in sensitivity. Furthermore, the data of diagnosis in extreme age groups, small children and the elderly, is still limited.

For diagnosis, Polymerase Chain Reaction (PCR) or the use of a serological method that can demonstrate a fourfold increase in an antibody titer between bleeds are regarded as the gold standard methods for diagnosis of dengue infection. However, both techniques are complicated, costly, and not practical for resource-limited settings. Detection of dengue NS1 antigen and dengue-specific IgM by point-of-care rapid diagnostic test (RDT) have been widely used as the main diagnosis method in clinical practice, especially in remote and resource-limited areas [2,3]. Unfortunately, the limited sensitivity restricts their diagnostic capacity. The meta-analysis results revealed that RDT dengue NS1 had a sensitivity of only 66% (95% CI: 54–81%) with a specificity of 99% (95% CI: 96–100%) [4]. The limitations of NS1 detection in clinical practice include a sensitivity decrease for secondary infections and during later disease stages (≥ day 5 of onset), whereby viremia levels are lower [3].

Dengue IgM starts to increase on Day 4 after the onset of illness [2]. However, the low sensitivity during the febrile/convalescent phase limits the potential usefulness for clinical diagnosis and management. Dengue IgM by enzyme-linked immunosorbent assay (ELISA) conducted during the febrile phase yielded a detection of 50%, while secondary infection cases have lower IgM titers [5]. The RDT for dengue IgM revealed a sensitivity of 53.5% and specificity of 100% in clinical specimens, while the combination of dengue NS1 and IgM detection increases the sensitivity of RDT for dengue diagnosis to a rate of 88.7% [6].

We hypothesize that the lower amount of dengue NS1 antigen or antibodies (IgM and IgG) against dengue virus particles during the early stages of infection might result in the lowering of diagnostic test sensitivity. For this reason, increasing the concentration of the target antigen or antibody in the clinical specimens might increase the efficiency of the diagnostic test. Several methods can be applied to increase the concentration of antigens or antibodies, such as precipitation methods using salt (ammonium sulfate), organic solvent (ethanol, acetone), or acid (trifluoroacetic acid) [7,8]. However, these methods require several steps and consume considerable time. Ultrafiltration using a molecular weight cut-off (MWCO) membrane, another method to concentrate the protein, can increase the target of analytes for point-of-care testing [9]. In addition, Russel et al. successfully used the ultrafiltration technique to concentrate Japanese encephalitis and dengue-virus-like particles in the supernatant to a final concentration of 450 times [10]. Therefore, in this study, we aimed to concentrate dengue NS1 antigen and antibodies by using the ultrafiltration technique in order to enhance the sensitivity of dengue NS1 and antibody detection assays in clinical sera samples. Furthermore, the diagnostic performance of various diagnostic approaches is described.

## 2. Materials and Methods

### 2.1. Clinical Specimens

The acute sera from adult patients who have received PCR confirmation for dengue (n = 51) and non-dengue febrile illness (n = 35) (dengue PCR negative with other conformational diagnoses) patients were selected from the previous cohort of enrolled acute undifferentiated febrile illness (AUFI) patients, aged ≥ 15 years, at the Hospital for Tropical Diseases (HTD), Bangkok, Thailand [11]. The definite diagnoses of dengue and other tropical infections were confirmed through positive PCR results, hemocultures, or seroconversion of standard serology at HTD, as reported previously [11]. All sera were kept at −80 °C at HTD and thawed specifically for this experiment.

The sample size was informed by results of a previous study that reported the sensitivity of RDT dengue NS1 to be 66% [4] and by our assumption that the concentration method could improve the sensitivity of the method to 85%. The sample size was, therefore, calculated with a desired assay performance of 85% sensitivity, 90% specificity, and 10% acceptable error. Using a sample size calculation formula for estimating sensitivity and specificity with alpha error at 0.05 [12], the sample size required was 49 for PCR-positive dengue samples and 35 for PCR-negative AUFI samples.

### 2.2. Quantitative Reverse Transcription Polymerase Chain Reaction (qRT-PCR)

Viral RNA was extracted from 200 μL of serum specimens using a QIAamp viral RNA mini kit (Qiagen, Hilden, Germany) according to the manufacturer’s instructions. The viral RNA was eluted in 50 μL of nuclease-free water and stored at −80 °C until analysis. The DENV serotyping was detected by Quantitative Reverse Transcription Polymerase Chain Reaction (qRT-PCR) kit (Sacace Biotechnologies, Como, Italy) on the CFX96 Touch Real-Time Detection System (Bio-Rad) using previously described primer pairs and protocols [13].

### 2.3. Serology

The commercial ELISA of anti-Dengue Virus Type 1–4 IgG, IgM, and Dengue Virus NS1 were performed according to manufacturer instructions (EUROIMMUN, Lübeck, Germany) [14]. In brief, the serum samples of the IgG and IgM tests were diluted at 1:101 in the sample buffer, while the serum samples of the NS1 test were diluted at 1:20 in the sample buffer. The IgG, IgM, and NS1 mixtures were added into 96-well microtiter plates containing the purified dengue virus antigens and monoclonal anti-dengue virus NS1 coated antibodies, respectively. The reaction was allowed to incubate for 60 min at 37 °C. The wells were subsequently washed three times using a working strength wash buffer for each wash. A conjugate incubation process for IgG, IgM, and NS1 was applied by adding peroxidase-labeled anti-human IgG, IgM, and anti-Dengue virus NS1 antibodies, respectively. The microtiter plates were washed to remove unbound specific binding prior to staining with chromogen or substrate solution into each of the microtiter plates for 15 min. Finally, the reaction was stopped by adding 0.5 M sulfuric acid, and the photometric measurement of the color intensity was performed at a wavelength of 450 nm and at a reference wavelength of 620 nm. The IgG, IgM, and NS1 results were reported semi-quantitatively by calculating a ratio of the extinction value of the control or patient sample over the extinction value of the calibrator. A specimen was considered positive if the ratio was ≥1.1, borderline if the ratio was ≥0.8 to <1.1, and negative if the ratio was <0.8. The borderline ELISA results were interpreted and analyzed as negative. The sensitivity of NS1, anti-Dengue IgM, and IgG ELISA were 100%, 38.1%, and 94.3%, while the specificity values were 99.2%, 100%, and 98.5%, respectively [14].

For RDT, careUS^TM^ Dengue Combo NS1 & IgM/IgG Kit (WellsBio, Seoul, Korea) was applied according to manufacturer instructions [15]. In brief, 60 µL and 10 µL of serum were applied for NS1 and IgM/IgG detection, respectively. Sequentially, 1 drop (approximately 35 µL) of assay buffer was added into the IgM/IgG sample well. All tests were conducted by an experienced blinded operator and data were collected between 15–20 min after assay initiation. The previous report sensitivity of RDT for NS1, IgM, and IgG were 79.8%, 89.1%, and 82.6%, respectively, while the specificity values of all tests were 100% [15].

### 2.4. Concentration Method

The serum was three times concentrated by ultrafiltration technique using a centrifugal filter device with MWCO 10 kDa (Amicon^®^, Sigma-Aldrich, Burlington, MA, USA) according to manufacturer instructions. In brief, 300 ul of serum was applied and centrifuged at 10,000× *g* for 20 min at 4 °C. After centrifugation, approximately 100 uL of concentrated sera was collected for further analysis with a pipette.

### 2.5. Ethical Approval

The study was approved by the Ethics Committee of the Faculty of Tropical Medicine, Mahidol University (MUTM 2021-082-01). All participants signed consent in the original cohort to allow leftover specimen usage for related research.

### 2.6. Statistical Analysis

Data were validated, cleaned, and analyzed using StataBE v17.0 software (StataCorp, College Station, TX, USA). Characteristics of subjects were compared between those with dengue PCR-positive samples and those with dengue PCR-negative samples. Chi-square or Fisher’s Exact test was used for the comparison of categorical data. Diagnostic parameters, including sensitivity, specificity, positive predictive values, negative predictive values, positive likelihood ratio, and negative likelihood ratio, as well as their 95% confidence intervals, were determined for different diagnostic methods (pre- and post-concentration), using PCR results as the gold standard. In addition, the sensitivity was determined by the day of illness. Characteristics of patients with false negative RDT dengue NS1 and IgM were explored. The false negative cases were defined as patients who were dengue PCR-positive but who had negative test results for both RDT dengue NS1 and IgM. *p*-values < 0.05 were considered statistically significant.

## 3. Results

### 3.1. Characteristics of the Study Population

The study population consisted of 51 PCR-confirmed dengue infections and 35 non-dengue febrile illness patients. Demographic and clinical data of all selected patients are shown in Table 1. There were no differences in age, sex, or underlying diseases. On the day of illness, dengue patients visited the hospital earlier than non-dengue illness patients (Table 1). The Tourniquet test was positive (≥10 points) more often in the dengue group. There were a total of 40 DF and 11 DHF cases. The diagnoses of non-dengue febrile illnesses included bacteremia, influenza, leptospirosis, and murine typhus.

### 3.2. Diagnostic Performance of Tests

The PCR results were used as the gold standard for diagnostic performance analysis of both ELISA and RDT.

#### 3.2.1. ELISA and RDT: Pre-Concentration (Pre-Con) Specimens

Sera were subjected to ELISA testing for detection of NS1, IgM, and IgG. NS1 had the highest sensitivity of 82.4%, with 94.3% specificity (Table 2). The IgM detection showed low sensitivity at 27.5%. Although for IgG, the test showed high sensitivity (86.3%), its very low specificity (5.71%) limits its utility in the diagnosis of acute dengue infection (Table 2).

The RDT was used to test the samples for NS1, IgM, and IgG. A low IgM sensitivity of 17.9% was obtained (Table 2). The combined analysis of RDT NS1 and IgM yielded a sensitivity of 78.4% and a specificity of 97.1% (Table 2).

#### 3.2.2. ELISA and RDT: Post-Concentrated (Post-Con) Specimens

Sera were concentrated by volume approximately three times (3X) using the ultrafiltration technique. Only IgM and IgG were detected in the concentrated sera using ELISA.

**Figure 1:**
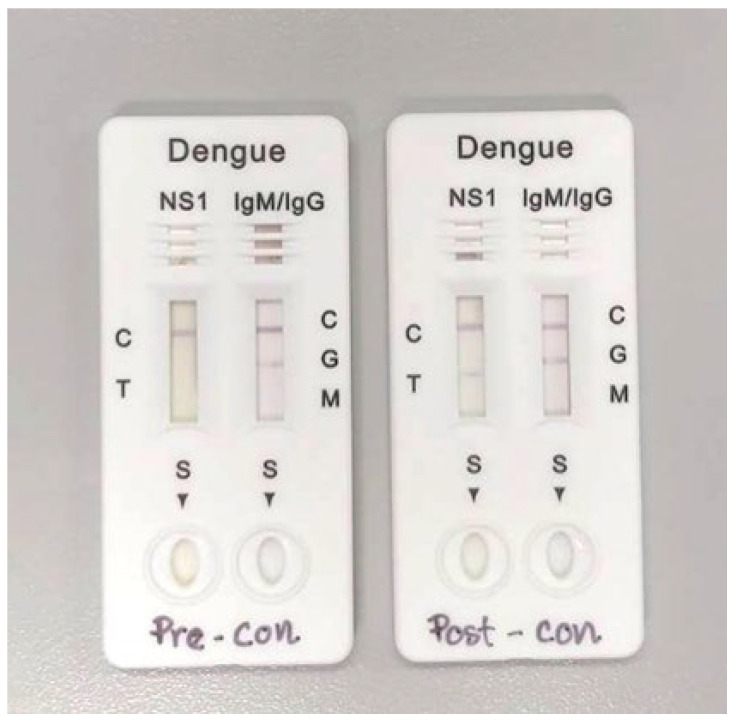
Example of RDT Pre-concentration and Post-concentration results.

The RDT was used to detect NS1, IgM, and IgG. Figure 1 displays the example of RDT Pre-con and Post-con results. Results showed that the concentration method yield slightly improved sensitivity of RDT-NS1 from 76.5% to 80.4%, with 100% specificity (Table 2). Unexpectedly, antibody detection (both IgM and IgG) showed decreased sensitivity but increased specificity after sample concentration (Table 2). By combining the antigen (NS1) and antibody (IgM) approach, the concentration method improved sensitivity from 78.4 to 82.4% and specificity from 97.1 to 100% (Table 2). The diagnostic performance of Post-con RDT-NS1 and RDT-IgM was comparable to those of ELISA-NS1.

The likelihood ratios were also calculated for different tests. The likelihood ratio indicates how likely it is that the patient has the disease after the test. For positive likelihood ratio, the larger the value is above 1 suggests the increased likelihood of disease after testing positive. For negative likelihood ratio, values closer to zero suggest a decreased likelihood of disease after testing negative. The combined analysis of RDT-NS1 and -IgM yielded the positive likelihood ratio of 27.5, negative likelihood ratio of 0.22, and 0.18 for pre- and post-concentration methods.

#### 3.2.3. ELISA and RDT Sensitivity by Day of Onset of Illness: Pre- and Post-Con Specimens

**Figure 2:**
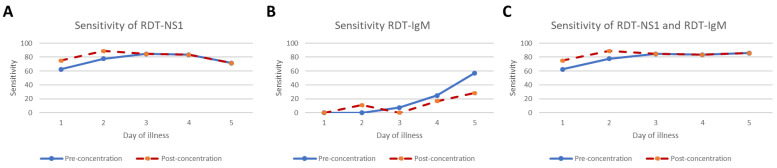
The sensitivity of dengue RDT for NS1 (**A**), IgM (**B**) and combined NS1 and IgM (**C**) by day of illness for both pre-and post- concentration are plotted in the graph. The sensitivity on Day 6 was not estimated due to the small sample size.

Further analysis was conducted to study the sensitivity of different dengue diagnostic methods by day of illness, as shown in Table 3. The sensitivity values of dengue RDT by day of illness for both pre- and post- concentration are plotted in Figure 2. Interestingly, the concentration method could increase the sensitivity of dengue infection diagnosis only during very early stages, such as Days 1–2 of onset of illness (Figure 2A,C). 

### 3.3. The Results of False Negative RDT on NS1 Ag and DEV IgM

We also focused on characteristics of cases with Pre-con false negative RDT-NS1 and RDT-IgM which might have been missed during clinical practice screening, as shown in Table 4. We found a trend that patients who came for diagnosis during the early stages of the disease, especially on Day 1 of illness, had a high rate of false negativity when compared with patients who visited the hospital on the Days 3–5 of illness. Furthermore, dengue serotype 3 (determined by qRT-PCR) had the highest rate of false negativity. Interestingly, the Tourniquet test was positive in 3 of 11 false negative RDT-NS1 and RDT-IgM cases.

## 4. Discussion

We described the diagnostic performance of different dengue diagnostic methods and conducted a preliminary trial to concentrate dengue antigens and antibodies by implementing the ultrafiltration technique. Three-time concentration by ultrafiltration method in conjunction with the combined analysis of NS1 and IgM could increase RDT diagnostic performance to a level comparable to ELISA-NS1. This method requires a reasonable amount of additional time and cost. Interestingly, the increase in sensitivity by the concentration method was promising during the very early stages of infection (Days 1–2 of illness).

The comparative diagnostics of dengue (Pre-con results) in this study echoed the limitations of dengue diagnosis in the febrile phase in clinical practices. Firstly, the moderate sensitivity of RDT-NS1 was comparable to previous reports [4,16], although sera in the febrile phase of acute dengue infection were used. The sensitivity of RDT-NS1 in this study was at peak levels on Days 3–4 of illness, which was different from a previous study whereby peak sensitivity was on Day 1 of illness [17]. With a 100% specificity and 100% PPV of RDT-NS1, this technique is very useful in dengue diagnosis in the febrile phase. Secondly, ELISA-NS1 showed the best diagnostic performance in terms of sensitivity and specificity [18,19]; however, ELISA is not practical as a point-of-care testing method. Thirdly, the sensitivity for IgM detection in the febrile phase of dengue infection was low. This study revealed very low sensitivity for IgM detection (27.5% by ELISA and 17.9% by RDT) when compared with the 40.3% which was previously reported in the meta-analysis [20]. This might be from using sera from the very early phases of dengue infection and the high rate of secondary dengue infection, which results in lower levels of IgM. The other possibility is that the standard ELISA we used had limited sensitivity, as mentioned in Materials and Methods. The high prevalence of previous dengue infection (positive dengue IgG) in adults in endemic areas, including Thailand [21], limits IgG utility in the diagnosis of acute dengue infection. Lastly, a combined analysis of the NS1 and IgM approach was suggested for diagnostics of acute dengue infection in clinical practices [6,20,22].

Specimen preparation techniques have been a challenging approach to enhancing the diagnosis of dengue infection. Previous studies have reported the successful enhancement of dengue viral antigens detection by specimen preparation techniques. Chen and colleagues applied a low pH glycine buffer treatment to serotype 2 dengue patients’ sera and revealed an increase in the detection rate of E and NS1 protein by ELISA testing [23]. Buonora and coworkers increased the ELISA-NS1 sensitivity from 47.8% to 57.7% by using heat dissociation in acute dengue serotype 4 patients [24]. Using ELISA and a single serotype might limit the application and generalization of the studies. Further studies in this field are required.

Protein ultrafiltration using MWCO membrane enables protein solution purification and increased sample concentration. It has been used to purify proteins, including antibodies and viruses [25,26]. However, it is not widely applied to medical purposes or in clinical practices. With commercial preparations, the ultrafiltration method is safe, reasonably priced (~USD 6 per test), and requires minimal additional diagnostics time (~20 min). This method requires only a centrifuge machine, equipment generally available in hospital laboratories. With similar sensitivities, the overall cost of RDT-NS1 with the ultrafiltration method was less when compared with ELISA- NS1, at USD 15 and USD 24, respectively. A slight increase in the diagnostic performance of NS1 detection was obtained through three-time concentration samples in this preliminary study. However, increased concentration, such as five-time concentration, can be applied to improve the test performance. Unfortunately, although this concentration method showed an increase in specificity, it decreased the sensitivity of both IgM and IgG detection. The reason of this occurrence is unknown but might be due to the prozone phenomenon of the antibody [27]. 

Of note, our study reported the highest rate of RDT-NS1 and RDT-IgM false negatives in serotype 3 (by qRT-PCR), whereas the previous study found the highest rate of false negativity NS1 in serotype 4 [28].

The meta-analysis revealed that the Tourniquet test, showing a pooled sensitivity of 58%, with 71% specificity, provided marginal benefits in the diagnosis of dengue infection [29]. However, this study showed positive results in 3 of 11 cases, whereby test results were negative according to both NS1 and IgM RDT. This result suggested some benefits of the classic clinical sign in the clinical diagnosis of acute dengue infection.

The study’s strengths include a combined analysis of RDT-NS1 and -IgM, which is practical for use in clinical practices, and an analysis of all dengue serotypes. However, the small sample size of the preliminary study limited the statistical power. Using a single manufacturer RDT might limit the generalization of the study. A future study with higher concentrations and larger sample size is warranted. Additionally, the study was performed by using only adult sera; the results may not be valid for extrapolation in small children.

## 5. Conclusions

We preliminarily improved diagnostic performances of acute dengue infection by using the ultrafiltration method. With a reasonable amount of additional time and cost, the three-time concentration method could slightly improve the sensitivity and specificity of combined RDT-NS1 and RDT-IgM analyses, especially in the very early stages of infection. Future studies are required.

## Figures and Tables

**Table 1:** Demographic and clinical characteristics of subjects.

		Total		Dengue PCR-Positive		Dengue PCR-Negative		*p*-Value
		**N**	**(%)**	**N**	**(%)**	**N**	**(%)**	
Total		86		51		35		
Sex								
	Female	34	39.5	21	41.2	13	37.1	0.707
	Male	52	60.5	30	58.8	22	62.9	
Age Group								
	15–30	40	46.5	28	54.9	12	34.3	0.083
	31–45	24	27.9	14	27.5	10	28.6	
	46–61	22	25.6	9	17.6	13	37.1	
Day of Illness								
	1–3	44	51.2	30	58.8	14	40.0	0.001
	4–6	33	38.4	21	41.2	12	34.3	
	7–11	9	10.5	0	0	9	25.7	
Tourniquet test								
	Not performed	17	19.8	14	27.5	3	8.6	0.001
	Negative	46	53.5	19	37.3	27	77.1	
	Positive	23	26.7	18	35.3	5	14.3	
Dengue Serotype *								
	1			14	27.5			
	2			9	17.6			
	3			14	27.5			
	4			14	27.5			
Final Diagnosis *								
	DF			40	78.4	0	0	
	DHF			11	21.6	0	0	
	Bacteremia			0	0	5	14.3	
	Influenza			0	0	10	28.6	
	Leptospirosis			0	0	10	28.6	
	Murine typhus			0	0	10	28.6	

* The percentages do not add up to 100% due to rounding.

**Table 2:** Summary of diagnostic performance of different techniques (N = 86).

	Sensitivity (95% CI)		Specificity (95% CI)		PPV (95% CI)		NPV (95% CI)		Positive LR (95%CI)		Negative LR (95%CI)	
	Pre-Concentration	Post-Concentration	Pre-Concentration	Post-Concentration	Pre-Concentration	Post-Concentration	Pre-Concentration	Post-Concentration	Pre-Concentration	Post-Concentration	Pre-Concentration	Post-Concentration
**ELISA-NS1**	82.4 (69.1–91.6)		94.3 (80.8–99.3)		95.5 (84.5–99.4)		78.6 (63.2–89.7)		14.4 (3.73–55.7)		0.19 (0.10–0.34)	
**RDT-NS1**	76.5 (62.5–87.2)	80.4 (66.9–90.2)	100.0 (90.0–100.0)	100.0 (90.0–100.0)	100.0 (91.0–100.0)	100 (91.4–100.0)	74.5 (59.7–86.1)	77.8 (62.9–88.8)	NA	NA	0.24 (0.14–0.39)	0.20 (0.11–0.34)
**ELISA-IgM**	27.5 (15.9–41.7)	23.5 (12.8–37.5)	91.4 (76.9–98.2)	97.1 (85.1–99.9)	82.4 (56.6–96.2)	92.3 (64.0–99.8)	46.4 (34.3–58.8)	46.6 (34.8–58.6)	3.2 (0.99–10.3)	8.24 (1.12–60.5)	0.79 (0.65–0.97)	0.79 (0.67–0.93)
**RDT-IgM**	17.9 (8.4–30.9)	11.8 (4.44–23.9)	97.1 (85.1–99.9)	100.0 (90.0–100.0)	90.0 (55.5–99.7)	100.0 (54.1–100.0)	44.7 (33.3–56.6)	43.8 (32.7–55.3)	6.18 (0.82–46.6)	NA	0.85 (0.74–0.97)	0.88 (0.80–0.98)
**ELISA-IgG**	86.3 (73.7–94.3)	86.3 (73.7–94.3)	5.71 (0.7–19.2)	5.71 (0.7–19.2)	57.1 (45.4–68.4)	57.1 (45.4–68.4)	22.2 (2.8–60.0)	22.2 (2.8–60.0)	0.92 (0.80–1.05)	0.92 (0.80–1.05)	2.4 (0.53–10.9)	2.4 (0.53–10.9)
**RDT-IgG**	66.7 (52.1–79.2)	62.7 (48.1–75.9)	25.7 (12.5–43.3)	34.3 (19.1–52.2)	56.7 (43.2–69.4)	58.2 (44.1–71.3)	34.6 (17.2–55.7)	38.7 (21.8–57.8)	0.90 (0.68–1.18)	0.96 (0.69–1.31)	1.3 (0.65–2.57)	1.09 (0.61–1.94)
**RDT-NS1 and RDT-IgM**	78.4 (64.7–88.7)	82.4 (69.1–91.6)	97.1 (85.1–99.9)	100.0 (90.0–100.0)	97.6 (87.1–99.9)	100.0 (91.6–100.0)	75.6 (60.5–87.1)	79.5 (64.7–90.2)	27.5 (3.96–190.0)	NA	0.22 (0.13–0.38)	0.18 (0.10–0.32)

PPV = Positive Predictive Value; NPV = Negative Predictive Value; LR = Likelihood Ratio; NA = Not available due to zero value in the denominator (1-specificity = 0).

**Table 3:** Sensitivity of different dengue diagnostic methods by day of illness.

Sensitivity		Day of Illness	
		1–3 N = 44 Sensitivity (95% CI)	4–6 N = 33 Sensitivity (95% CI)
ELISA-NS1			
	Pre-concentration	83.3 (65.3–94.4)	81.0 (58.1–94.6)
	Post-concentration	-	-
RDT-NS1			
	Pre-concentration	76.7 (57.7–90.1)	47.6 (25.7–70.2)
	Post-concentration	83.3 (65.3–94.4)	76.2 (52.8–91.8)
ELISA-IgM			
	Pre-concentration	13.3 (3.8–30.7)	47.6 (25.7–70.2)
	Post-concentration	16.7 (5.64–34.7)	33.3 (14.6–57.0)
RDT-IgM			
	Pre-concentration	3.33 (0.08–17.2)	38.1 (18.1–61.6)
	Post-concentration	3.33 (0.08–17.2)	23.8 (8.22–47.2)
ELISA-IgG			
	Pre-concentration	83.3 (65.3–94.4)	90.5 (69.6–98.8)
	Post-concentration	83.3 (65.3–94.4)	90.5 (69.6–98.8)
RDT-IgG			
	Pre-concentration	56.7 (37.4–74.5)	81 (58.1–94.6)
	Post-concentration	53.3 (34.3–71.7)	76.2 (52.8–91.8)
RDT-NS1 and RDT-IgM			
	Pre-concentration	76.7 (57.7–90.1)	81.0 (58.1–94.6)
	Post-concentration	83.3 (65.3–94.4)	81.0 (58.1–94.6)

**Table 4:** Characteristics of patients with false negative RDT-NS1 and RDT-IgM.

		Dengue PCR-Positive				
		RDT-Positive		RDT-Negative		*p*-Value
		N	(%)	N	(%)	
Total		40		11		
Sex						
	Female	18	45.0	3	27.3	0.328 *
	Male	22	55.0	8	72.7	
Age Group						
	15–30	22	55.0	6	54.5	0.525 *
	31–45	12	30.0	2	18.2	
	46–61	6	15.0	3	27.3	
Day of Illness^†^						
	1	5	12.5	3	27.3	0.698 *
	2	7	17.5	2	18.2	
	3	11	27.5	2	18.2	
	4	10	25.0	2	18.2	
	5	6	15.0	1	9.1	
	6	1	2.5	1	9.1	
Tourniquet Test						
	Not performed	12	30.0	2	18.2	0.513 *
	Negative	13	32.5	6	54.5	
	Positive	15	37.5	3	27.3	
Dengue Serotype ^†^						
	1	12	30.0	2	18.2	0.183 *
	2	9	22.5	0	0	
	3	9	22.5	5	45.5	
	4	10	25.0	4	36.4	
Final Diagnosis						
	DF	30	75.0	10	90.9	0.418 *
	DHF	10	25.0	1	9.1	
Serology						1.00
	Primary	1	2.6	0	0	
	Secondary	38	97.4	11	100	

* Fisher’s exact test. ^†^ The percentages do not add up to 100% due to rounding.

## Data Availability

The dataset can be requested from the corresponding author.
